# Supplementary material for: Are carbon emissions trading and green financial instruments synergistic? -Comprehensive quantitative research based on content analysis
Source: PLoS One. 2024 Mar 7;19(3):e0298601. doi: 10.1371/journal.pone.0298601 (PMC10919601; doi:10.1371/journal.pone.0298601)
Supplement: S1 Data — (ZIP) [file pone.0298601.s001.zip › Data/地级市Z_F工作报告.zip!/╒π╜¡/╝╬╨╦╩╨/└┤╫╘╣1⁄2╓┌║┼í╛┬φ┐╦╩2╛▌═°í┐_2016.docx]

各位代表：

　　现在，我代表市人民政府向大会报告工作，请予审议，并请市政协委员和其他列席人员提出意见。

　　一、“十二五”发展回顾

　　“十二五”时期是嘉兴发展进程中不平凡的五年。面对严峻复杂的国内外环境，市政府全面贯彻省委、省政府决策部署，在市委的正确领导下，全力以赴稳增长、调结构、促改革、治环境、惠民生，较好地完成了“十二五”规划确定的预期目标，经济社会发展迈上了新台阶。

　　这五年，是综合实力稳步增强的五年。地区生产总值从2315.5亿元增加到3517.1亿元，年均增长8.6%，人均地区生产总值达到1.23万美元。财政总收入、一般公共预算收入达到638.8亿元和350.4亿元，分别是2010年的1.9倍和2倍。金融机构本外币存贷款余额达到5957亿元和4920亿元，分别是2010年的1.7倍和1.8倍。累计完成固定资产投资9775.7亿元，是“十一五”时期的1.8倍，嘉绍通道、钱江通道等一批重大工程建成并投入运行。浙商回归到位资金981.5亿元，连续四年位列全省同档城市第一。

　　这五年，是转型升级步伐加快的五年。科技创新能力不断增强，研究与试验发展经费支出占地区生产总值比重从2.1%提高到2.7%，累计引进各类高层次人才3.6万名，其中国家、省“千人计划”专家各111名，新增发明专利授权2985项。产业结构不断优化，规模以上工业总产值突破7500亿元，战略性新兴产业、高新技术产业、装备制造业占规模以上工业增加值比重分别达到44%、42.3%和24.9%；服务业增加值突破1500亿元，年均增长10.1%，高于地区生产总值增速1.5个百分点；粮食总产量保持在122万吨左右。三次产业结构从5.5∶57.7∶36.8调整为4.0∶52.6∶43.4。资源节约集约利用取得积极进展，累计腾退低效用地4.6万亩，单位生产总值能耗比2010年下降19.3%，省下达的主要污染物减排目标全面完成。

　　这五年，是改革开放不断深化的五年。新型城镇化、“多规合一”等改革获批国家试点，在全省率先启动资源要素差别化配置、行政审批层级一体化、综合行政执法等改革，五年累计获批国家、省改革试点146项。编制政府权力清单和责任清单，市级部门行政权力从11552项减少到3875项，428项审批事项下放至县（市、区），市级部门行政审批事项减少到59项，全面取消非行政许可审批事项，落实事中事后监管制度517项；新增市场主体26.2万家，境内外上市公司达到40家。对外开放取得新进展，累计实现进出口总额1538.1亿美元，其中出口1069.6亿美元，实际利用外资108.9亿美元。嘉兴出口加工区升格为国家综合保税区，嘉兴港口岸扩大开放获得国家批复。世界互联网大会永久落户乌镇。

　　这五年，是城乡建设加快推进的五年。综合交通体系不断完善，路网密度达到206.6公里/百平方公里，内河航道通航里程达到1957公里，“三纵三横三连”高速公路网基本建成。资源能源保障能力进一步增强，新增110千伏及以上变电容量1270万千伏安，自来水日供水能力达到249.4万吨，累计供应天然气18.2亿立方米。市域一体化发展取得积极进展，“1640”、“四百一千”的现代化网络型田园城市框架初步形成，累计实施城市有机更新2.5万亩，完成土地复垦5.1万亩，改造集聚农房9.2万户，常住人口城镇化率由53.3%提高到60.9%，统筹城乡发展水平位居全省首位。

　　这五年，是生态环境持续改善的五年。水环境质量实现历史性转变，累计完成黑臭河和垃圾河整治1893公里，新建污水收集管网2389公里，8847家工业企业实现污水全入网，市控以上断面水质从五类、劣五类为主转变为四类、三类为主，四类水以上比重由18.7%提高到77.6%，交接断面水质考核从不合格转变为连续优秀。生猪养殖业减量提质取得重大突破，生猪存栏量从294.7万头削减至32.8万头。大气污染整治深入实施，超额完成燃煤锅炉、黄标车淘汰任务，空气质量平均优良率达到64.5%。城乡面貌明显改善，累计拆除违法建筑4738万平方米，改造旧住宅区、旧厂区、城中村6731万平方米，新建改造绿化22万亩，建成生态绿道851公里。新增国家级生态镇（街道）34个。

　　这五年，是群众得到更多实惠的五年。各级财政民生支出累计达到1187.5亿元，年均增长18.2%。城镇居民、农村居民人均可支配收入分别达到45499元、26838元，农村居民收入连续12年位居全省第一，城乡居民收入差距缩小到1.695∶1。五年新增城镇就业人口48万，城镇登记失业率控制在2.9%以内。基本养老、基本医疗保险参保人数分别达到226万人和197万人，城乡低保标准统一提高到664元，最低月工资标准由980元提高到1660元。义务教育入学率和巩固率达到100%，高中段教育入学率达到99.1%，高等教育毛入学率达到58%，“十二五”期间所有县（市、区）全部通过国家义务教育均衡发展评估。国家公共文化服务体系示范区创建成效明显，城乡图书馆、文化馆总分馆建成率100%。养老机构床位增加到2.9万张，是2010年的3倍。成功创建并蝉联全国文明城市，实现省级平安市、县（市、区）创建“十一连冠”、“满堂红”。

　　这五年，是政府自身建设不断加强的五年。“三公”经费预算全部公开，公务考察、接待和用房管理进一步规范。自觉接受市人大及其常委会监督和市政协民主监督，累计办理市人大代表建议1644件、市政协提案1986件。法治政府建设取得新进展，制定出台政府重大行政决策程序规定，决策科学化民主化法治化水平得到提高。累计清理政府规范性文件1199件。

　　过去一年，市政府按照市七届人大五次会议确定的各项目标任务，主动适应经济发展新常态，牢牢把握稳中求进的工作总基调，坚持以提高经济发展质量和效益为中心，统筹推进经济社会发展，全年实现地区生产总值3517.1亿元，增长7%；完成财政总收入638.8亿元，其中一般公共预算收入350.4亿元，分别增长8.6%和7.1%；城镇和农村居民人均可支配收入分别增长8%和8.8%。重点抓了以下五个方面的工作：

　　一是全力以赴稳增长。组织实施“项目大推进、招商大引进、服务大跟进”专项行动，实现固定资产投资2513.8亿元，增长13.2%。开展创新驱动发展拓市场试点，对出口额在400万美元以下的生产型企业实行出口信用保险政府联保，实现外贸出口229.3亿美元。海宁皮革城列入国家市场采购贸易方式试点。大力发展电子商务，社会消费品零售总额1494.6亿元，增长11%，网络零售额增长36.8%。组织开展“转型发展服务年”活动，设立总额59亿元的政府性产业基金，减免企业税费97.1亿元，新增制造业贷款43.4亿元。实施小微企业三年成长计划，新增“个转企”1669家、新三板挂牌企业19家、上市公司5家。开展金融风险防控专项整治，企业应急转贷资金制度实现县（市、区）全覆盖。

　　二是坚定不移推进转型升级。新引进大院名校共建创新载体29家，新增省级企业研究院17家，新认定高新技术企业108家。嘉兴科技城成为全省五大科技平台之一，秀洲高新技术产业园区升格为国家级高新技术产业开发区。市农科院水稻新品种选育成果获得国家科技进步二等奖。深入实施“四换三名”工程，完成“机器换人”投入538.5亿元，腾退低效用地1.2万亩，整治提升低端落后企业2392家。制定实施加快楼宇经济提质发展三年行动计划。中德、中日、中荷、中法等4家产业园区被列为首批省级国际产业合作园，南湖基金小镇、嘉善巧克力甜蜜小镇、海盐核电小镇、海宁皮革时尚小镇和桐乡毛衫时尚小镇入选全省首批特色小镇。

　　三是持续推进各项改革。深化“四张清单一张网”改革，建立完善部门权力清单、责任清单动态调整机制，实现镇（街道）权力清单、责任清单全覆盖。深化行政审批层级一体化改革，启动相对集中行政许可改革试点。实施综合行政执法改革，组建市、县、镇三级综合执法机构。推进商事制度改革，全面实行“五证合一、一照一码”登记。组织开展国家深化工业用地市场化配置改革试点，建立全省首家市级资源要素交易中心。嘉善、海宁被列为国家县城深化基础设施投融资体制改革试点。积极构建供销、生产、信用合作“三位一体”农民合作经济组织体系，推进土地承包经营权、宅基地使用权、集体经济股权确权颁证，县、镇两级农村产权交易中心实现全覆盖。

　　四是加大城乡建设和环境整治力度。启动国家新型城镇化综合试点，扎实推进“多规合一”，完成嘉兴空间发展与保护总体规划编制。深入实施“三改一拆”，组织开展“公铁”沿线环境整治，拆除各类违法建筑1458.3万平方米，拆出土地1299万平方米，拆后利用率达到77.2%。获批国家海绵城市建设试点城市。加大交通治堵力度，中心城区新增公共停车位588个、公交专用道13.3公里，打通由拳路、商务大道等5条断头路。坚定不移打好治水攻坚战，对全市所有城镇污水处理厂实施提标改造，新增污水达标入网企业2121家、农村生活污水治理受益农户13.5万户。加快平湖塘延伸拓浚、扩大杭嘉湖南排两大水利骨干工程建设，完成市域外配水工程（杭州方向）项目省级立项。大力实施“五气共治”，淘汰燃煤锅炉1603台、黄标车1.8万辆。严格执行新环保法，查处环境违法案件1105件。

　　五是千方百计增进民生福祉。健全就业促进机制，帮助4.5万名城镇失业人员实现再就业。加快社会保障制度整合衔接，实现大病保险全覆盖。加大社会救助力度，发放困难群体医疗救助金5453.2万元。加强城乡住房保障，新开工建设保障性安居工程住房1.2万套。完善养老服务体系，居家养老服务照料中心城乡社区覆盖率分别达到100%和91%。深入推进教育领域综合改革，实施新一轮学前教育三年行动计划和特殊教育三年行动计划，启动中考招生制度改革，海宁、桐乡、海盐成为全省首批教育基本现代化县。深化公立医院综合改革，稳步推进“双下沉、两提升”，促进医疗卫生资源城乡均衡分布。推进公共体育设施免费开放，举办市第八届运动会暨第三届市民运动会。深入推进公共法律服务体系建设，镇（街道）公共法律服务中心实现全覆盖。强化社会风险排查化解管控，加强新居民管理服务，安全生产、食品药品安全形势总体平稳，社会保持和谐稳定。

　　各位代表，“十二五”胜利收官，成绩来之不易！这是省委、省政府和中共嘉兴市委正确领导的结果，是全市人民奋力拼搏的结果。在此，我代表市人民政府，向全市人民和外来建设者，表示衷心的感谢！向人大代表、政协委员，各民主党派、工商联、人民团体和社会各界人士，向驻嘉人民解放军和武警部队官兵，表示衷心的感谢！向所有关心和支持嘉兴发展的海内外朋友们，表示衷心的感谢！

　　我们既要看到成绩，也要看到前进中的困难和挑战。“十二五”期间，地区生产总值等个别指标未能达到预期目标。随着经济下行压力加大，实体经济发展面临更多困难；科技创新能力有待增强，传统产业提升不快、新兴产业占比不高；中心城市集聚辐射带动能力亟待提高；资源环境约束紧张，治水治气任重道远；医疗、养老、教育、交通等公共服务离广大人民群众的期望还有一定的差距；安全生产、社会治安、网络等领域存在潜在风险隐患，创新社会治理、维护社会稳定的压力不小。在政府自身建设方面，一些政府工作人员改革意识、责任意识、法治意识不强，庸政懒政怠政依然存在，消极腐败现象还时有发生。我们一定高度重视这些问题，切实采取更加有力措施认真加以解决。

　　二、关于《“十三五”规划纲要（草案）》的说明

　　“十三五”是充满机遇、充满挑战的五年，是嘉兴高水平全面建成小康社会的决胜阶段。根据中共嘉兴市委《关于制定嘉兴市国民经济和社会发展第十三个五年规划的建议》，市政府认真研究并听取各方面意见，制定了《嘉兴市国民经济和社会发展第十三个五年规划纲要（草案）》，提请大会审议。下面我就三个问题作简要说明：

　　（一）“十三五”发展的指导思想

　　《纲要(草案)》按照市委《建议》提出了“十三五”时期经济社会发展的指导思想：高举中国特色社会主义伟大旗帜，以马克思列宁主义、毛泽东思想、邓小平理论、“三个代表”重要思想、科学发展观为指导，深入贯彻习近平总书记系列重要讲话精神，以“四个全面”战略布局为统领，以创新、协调、绿色、开放、共享五大发展理念为引领，以“八八战略”为总纲，以“干在实处永无止境、走在前列要谋新篇”为新使命，以“更进一步、更快一步，继续发挥先行和示范作用”为总要求，坚持发展第一要务，坚持转型升级不动摇，紧扣提高经济发展质量和效益这一中心，加快形成引领经济发展新常态的体制机制和发展方式，统筹推进经济建设、政治建设、文化建设、社会建设、生态文明建设，加快“两富”“两美”嘉兴建设，高水平全面建成小康社会，全力打造现代化网络型田园城市，以党的诞生地各方面工作取得的优异成绩向建党100周年献礼。

　　坚持发展第一要务是时代的要求，关系改革开放和现代化建设全局。坚持转型升级不动摇，紧扣经济发展质量和效益这一中心是嘉兴“十三五”发展的必由之路。加快形成引领经济发展新常态的体制机制和发展方式，统筹推进经济建设、政治建设、文化建设、社会建设、生态文明建设是嘉兴实现转型发展的重要保障。《纲要(草案)》顺应新形势新阶段提出新目标新任务，既坚持谋划长远，体现战略性、前瞻性，又注重着眼当前，反映现实性、可行性；既坚持统筹兼顾，对经济社会转型发展作出全面部署，又聚焦围绕一些重点领域和关键环节，提出具体措施、专项工程和重大项目，做到抓住关键、突出重点、推动全局。

　　（二）“十三五”发展的主要目标

　　《纲要（草案）》按照市委《建议》提出的指导思想和总体目标，提出了包括经济发展、创新驱动、民生福祉、资源环境等四个方面的经济社会发展主要指标，总的目标：

　　——综合实力更强劲。经济保持中高速增长，产业迈向中高端水平，创新驱动发展的格局基本形成，地区生产总值年均增长7%以上，到2020年地区生产总值、人均地区生产总值、城镇居民人均可支配收入、农村居民人均可支配收入均比2010年翻一番，并力争快翻。

　　——生态环境更优美。到2020年，能源资源开发利用水平明显提高，主要污染物排放总量大幅减少，黑臭河和劣五类水体全面消除，在上游来水水质达标基础上，市控以上断面三类水成为主体，PM2.5平均浓度明显下降，空气质量优良天数比例达到75%以上，让嘉兴的天更蓝、地更净、水更清。

　　——城乡区域更协调。市域空间发展格局更加优化，城乡基础设施进一步完善，城乡公共服务均等、居民收入均衡、要素配置合理、产业发展融合的体制机制加快构建，“县域经济”向“城市经济”加快转型，到2020年常住人口城镇化率达到68%，统筹城乡发展继续走在前列。

　　——人民生活更幸福。社会就业更加充分，社会分配更加公平，公共服务体系更加完善，城乡居民人均可支配收入年均增长7%以上，五年新增城镇就业人口30万，到2020年新增劳动力平均受教育年限达到14年，人均期望寿命达到82.5岁，广大群众的富足感、获得感和幸福感进一步增强。

　　——治理体系更完善。重点领域及关键环节改革取得决定性成果，治理制度化、规范化、程序化、法治化、信息化水平不断提高，人民民主更加健全，法治政府基本建成，司法公信力明显提高，努力打造社会活力强、社会秩序优、社会风气正的地区。

　　（三）“十三五”发展的主要任务

　　《纲要（草案）》提出“十三五”发展必须认真践行创新、协调、绿色、开放、共享五大发展理念，并对今后五年我市经济社会发展作出了全面部署，提出了明确任务。主要是以下九个方面：

　　一是推动创新成为引领发展第一动力。把创新摆在发展全局的核心位置，不断推进科技创新、制度创新等各方面创新，加快构建有利于“大众创业、万众创新”蓬勃发展的政策制度环境和公共服务体系。创新人才发展体制机制，优化人才发展平台布局，着力打造人才强市。完善区域创新体系，高标准建设一批科技创新集聚区和“泛孵化器”，大力培育创新企业群。完善科技服务体系，推进科技金融紧密结合，全力打造长三角科技企业孵化高地和高科技成果转化重要基地。

　　二是构建现代产业发展新高地。突出制造业强市、服务业兴市不动摇，加快打造互联网经济强市，统筹推进战略性新兴产业、高新技术产业发展和传统优势产业改造提升。重点培育信息、文化、旅游、时尚、金融、节能环保、高端装备制造和新材料等八大千亿级产业，打造支撑嘉兴未来发展的新增长极。推动生产性服务业向专业化和价值链高端延伸、生活性服务业向精细化和高品质转变。加快发展都市型生态农业。

　　三是打造城乡区域发展一体化先行区。强化发展空间总体管控，科学布局城镇空间、农业空间、生态空间，着力构建“一核三带三区”市域生产力布局，完善“1640”、“四百一千”的现代化网络型田园城市发展架构。全面推进国家新型城镇化综合试点，提升中心城市首位度，强化副城综合实力，增强镇一级的集聚功能、生产功能、服务功能，加快推进小城镇建设，着力打造“美丽乡村”升级版。

　　四是构建江南水乡优美生态环境。坚持绿色惠民，严格实施主体功能区制度和生态立市战略。加大生态环境治理力度，全力打好生活污水、工业污水、农业面源污染治理攻坚战，确保所有工业企业污水实现达标入网，城乡生活污水处理率达到90%；以PM2.5防治为重点，深入实施大气污染防治行动计划。落实最严格的耕地保护制度，科学划定永久性生态用地，加快构筑生态安全屏障。推进资源节约集约高效利用，大力发展循环经济、低碳经济，促进生产生活方式绿色低碳。

　　五是再造开放引领新优势。大力发展更高层次的开放型经济，按照“两个无差别、三个更好”的要求，全面融入大上海都市经济圈，以交通基础设施对接为基础，推进与上海在产业发展、要素配置、公共服务、政策环境等领域的同城化。全面融入长江经济带建设，积极参与长三角城市群协同发展，提升区域合作水平。创新对外开放体制机制，加快对外贸易优化升级，提高利用外资质量水平。

　　六是创造安居乐业幸福生活。坚持共享发展理念，提高公共服务供给水平。统筹推进各类教育优质均衡发展。健全城乡均等就业创业服务体系，完善收入分配制度和劳动者报酬增长机制，大力推进低收入群体加快增收。进一步完善社会保险体系、社会救助福利体系和住房保障体系。深化医药卫生体制改革，优化医疗卫生资源配置，实施全民健身计划，增强养老康复服务设施供给能力，努力让全市人民拥有更好的教育、更高质量的就业、更满意的收入、更可靠的社会保障、更健全的健康服务和更高水平的养老服务。

　　七是提升城市文化软实力。自觉践行社会主义核心价值观，倡导“崇文厚德、求实创新”的嘉兴城市人文精神和“勤善和美”的当代嘉兴人共同价值观。大力发展文化事业，深化国家公共文化服务体系示范区建设，扶持优秀文化产品创作生产，激发文化创新创造活力。创新文化产业发展业态，到2020年全市文化产业增加值占地区生产总值比重达到7%。注重城市形象塑造，培育一批具有嘉兴地域特色的重大文化品牌，打造江南水乡文化传承典范。

　　八是提升基础设施现代化水平。以满足需求、适度超前为导向，加快推进“四网一路一空港”建设，统筹推进嘉兴南站综合交通枢纽建设，实现内外交通“无缝衔接”、各种交通方式“零距离”换乘。打造“智慧嘉兴”，深入推进光网城市建设，运用互联网创新城市治理。强化能源供应保障，推进水利设施现代化，加强城市地下空间开发利用，提升市政公用设施建设水平。

　　九是深化重点领域改革和社会治理。围绕优化调整政府与市场关系，加快推进要素市场、政府职能、社会组织、法治建设等基础性制度创新。进一步完善市场监管机制，创新行政管理服务体制。健全公平高效市场环境，深化投融资体制、要素配置差别化和国资国企改革，再创民营经济新优势。加强供给侧结构性改革，激励市场主体供给创新，推动供给与需求两端协同发力。构建社会多元治理新格局，健全立体化社会治安防控体系，切实保障社会公共安全，增强全民法治意识和法律素养，完善社会信用体系，着力建设“法治嘉兴”。

　　各位代表，未来五年的奋斗目标鼓舞人心，催人奋进。我们坚信，有市委的坚强领导，在全市人民的共同努力下，我市“十三五”规划的宏伟蓝图一定能够胜利实现！

　　三、2016年主要工作

　　起跑决定后程，首战影响决战。2016年是高水平全面建成小康社会决胜阶段的开局之年，务必要保持经济社会平稳健康发展。综合分析各方面因素，建议今年全市经济社会发展的主要预期目标为：地区生产总值增长7%-7.5%，一般公共预算收入增长6.5%，城镇居民人均可支配收入、农村居民人均可支配收入均增长8%，“三去一降一补”取得实质性进展，耕地保护和节能减排降碳指标完成省下达的目标任务。重点做好以下七项工作：

　　（一）努力保持经济稳定增长

　　着力扩大有效投资。更加注重投资的结构与效益，进一步加大产业投资、创新投资和重大基础设施投资力度，加快推进502个年度重点项目建设，确保固定资产投资增长11%，重大基础设施、重大产业、高新技术产业、生态环保项目投资增长15%。深入实施招商选资和浙商回归，力争引进世界500强公司、全球行业龙头企业和总投资1亿美元以上项目20个，实际利用外资达到25亿美元，浙商回归到位资金350亿元。创新投融资机制，推广政府与社会资本合作（PPP）模式，引导民间资本进入社会事业领域、参与重大项目建设。深入实施供而未用土地消化利用专项行动，切实保障大项目好项目用地需求。

　　着力提升产业竞争力。把发展实体经济放在更加突出的位置，实施《中国制造2025嘉兴行动纲要》，开展制造业“四化百项”工程，加快培育发展、改造提升智能制造装备、化工新材料、光伏新能源等“十大产业链”，推动工业做大做强。聚焦发展互联网经济，推进全国“新型智慧城市”标杆市试点和乌镇互联网创新发展试验区建设，启动培育10个互联网特色小镇、100个“互联网+”工业示范试点企业，力争信息产业产值突破1000亿元。全力办好第三届世界互联网大会。加快建筑业转型发展。突出服务业兴市，实施服务业投资“百项千亿”工程，大力发展现代物流、金融、科技信息等现代服务业，加强旅游资源和产业链整合，力争服务业增加值比重提高1.2个百分点。加快农业现代化步伐，深化农业“两区”建设，发展多种形式的适度规模经营，推动种养殖业转型升级，高度重视粮食安全，确保完成省下达粮食生产任务。高标准推进省级特色小镇建设，加快培育一批市级特色小镇，形成小镇建设联动发展、滚动推进的良好态势。

　　着力强化供给侧精准发力。健全企业优胜劣汰机制，突出整治“低、小、散”，对“僵尸企业”通过兼并重组、债务重组、破产清算实现市场出清。全年完成“个转企”1500家，新增股份制企业100家，实施兼并重组企业100家。切实为实体经济企业减负，对涉企成本进行全面清理、集中整治，大力帮扶企业化解资金链、担保链风险。加快房地产去库存、优结构，完善公积金政策，强化土地供应管理，保持房地产市场健康发展。优化金融供给结构，大力发展普惠金融，加强金融和金融衍生品监管，力争新增上市公司11家、新三板挂牌企业30家，直接融资规模达到500亿元。

　　（二）强化科技创新驱动

　　加强科技平台建设。高标准推进秀洲国家级高新技术产业开发区、嘉兴科技城建设，积极筹建浙大海宁（国际）研究院，推动省级高新技术园区实现县（市、区）全覆盖。打造众创、众包、众扶、众筹平台，构建企业、高校、科研机构、创客多方协同的新型创业创新机制，启动建设一批运行模式先进、配套功能完善的创业创新示范中心，力争新增市级以上科技孵化器11家，新建“两创中心”标准厂房60万平方米。

　　加速科技成果转化。深化产学研合作，组织实施一批重大科技应用示范专项，促进科技与产业融合。深入实施科技企业培育三年行动计划，力争新增高新技术企业150家、科技型企业400家，实现规模以上企业技术研发机构全覆盖。深化省科技金融改革创新试验区建设，扩大科技风险投资引导基金规模，撬动社会资本向科技创新领域聚集。加快网上网下科技大市场建设。

　　加快科技体制改革。启动省级科技创新改革试验区创建。健全多元化科技投入体系，探索科研项目经费、科技成果使用处置和收益管理创新，积极推行科技创新券制度，提高科技资金使用绩效。创新人才发展机制，深入实施“创新嘉兴·精英引领计划”，大力发展人力资源服务业，配套完善高层次人才亟需的国际教育、高端医疗等公共服务，力争新建院士专家工作站6家，新引育国家、省“千人计划”专家30名、市级领军人才100名。深入推进国家知识产权示范城市建设，力争新增发明专利授权850件。

　　（三）深入推进改革开放

　　积极推动各项改革实践。全力支持嘉善县域科学发展综合配套改革、平湖产业结构调整机制创新、海盐基本公共服务均等化、海宁循环经济示范城市、桐乡中小城市综合改革、秀洲分布式光伏发电应用示范区、南湖相对集中行政许可改革等国家、省改革试点，加快形成一批可复制、可推广的试点经验。积极推进财政预算管理改革，探索建立跨年度预算平衡机制，加强财政专项资金使用绩效评价。深化国资国企改革，实施市属经营性国有资产集中统一监管，加快建立外派监事会制度，推动从管资产向管资本转变。围绕“三权到人（户）、权随人（户）走”，深化土地承包经营权、宅基地使用权和集体收益分配权改革，积极稳妥推进农民住房财产权工作。

　　提升开放型经济引领水平。加快建设一批跨境电子商务产业园和示范企业，扩大高端机械、高新技术产品和服务贸易出口。支持外贸企业积极开拓国内市场。鼓励企业扩大先进技术、关键设备、节能环保等产品进口。加快嘉兴综合保税区建设步伐，高标准建设国际产业合作园。鼓励企业“走出去”，到境外建立生产基地、研发设计机构、能源资源基地、营销网络、“海外仓”，支持有条件的企业开展国际并购，实现产业链、价值链的全球布局。

　　全力推进接轨上海。推进基础设施互联互通，完成市域轨道交通线网与上海城市轨道交通网对接方案，加快沪乍杭铁路前期工作。大力推动沪嘉产业协同创新区建设，提升嘉善临沪产业合作园区、平湖张江长三角科技城和滨海新区等平台功能。充分复制借鉴上海自贸区改革经验，打造利用外资新高地。加强公共服务共建共享，大力引进上海高端教育、医疗资源，积极推进医保、公交“一卡通”应用，在沪嘉边界区域实现通讯同城化。建立健全接轨上海工作机制，加强市驻沪联络工作。

　　（四）努力建设美丽城乡

　　加快美丽城市建设。健全“多规合一”机制，进一步优化市域空间布局、功能定位。完善市域基础设施，加快推进杭州湾跨海大桥北接线二期、杭平申线航道改造等工程，继续推动嘉兴军民合用机场项目及市域轨道交通项目前期工作。加快中心城市国际商务区、湘家荡等重点区块开发建设，引导和支持各副中心城市特色化、差异化发展。深入实施城市有机更新，统筹城市地下空间开发利用和海绵城市建设，加强城市规划和设计，推进中心城区子城广场、南湖湖滨、博海路等片区更新开发。科学开展城市交通治堵，加快城市快速路网建设，新增公交专用道10公里。扩大市容市貌网格化管理覆盖面，健全“数字城管”应用体系，加大城市环境卫生顽疾的整治力度。

　　加快美丽新市镇建设。高品位、高质量推进新市镇规划建设，实施新一轮市级小城市培育三年行动计划，做好省级小城市试点申报。大力实施新市镇改造提升工程，推动城市基础设施和公共服务向城镇延伸，建成一批美丽示范街区和特色社区，提升新市镇的洁化绿化亮化美化水平。坚持产城融合，培育壮大新市镇主导产业。积极推动就地城镇化，促进有能力在城镇稳定就业和生活的农业转移人口在新市镇落户。

　　加快美丽乡村建设。深入推进美丽乡村四级联创，深化“百村示范、千村整治”工作。完善耕地保护补偿激励机制，深入推进农房改造建设，加快待整治自然村落向“四百一千”集聚，完成土地整治复垦7500亩。加强村庄建设规划和农房设计，加大传统村落民居和历史文化村落保护力度。实施新一轮村级集体经济壮大工程，鼓励发展民宿经济、农家乐和农村电商，推进富民强村。

　　（五）加强环境治理和保护

　　加大治水治气治土力度。持续深化“五水共治”，组织实施城乡生活污水治理、污泥固废整治、河湖清淤连通“三大工程”，新增受益农户5万户。加快城镇集中式污水处理厂扩容提标改造，开工建设城东再生水厂，力争市控以上断面四类及以上水质达到80%以上。完善生猪养殖业常态化监管机制，巩固减量提质成果。加快市域外配水工程（杭州方向）步伐，完成工程初步设计，同步开展分质供水前期工作。实施区域水系综合治理，加快推进平湖塘延伸拓浚、杭嘉湖扩大南排等水利工程建设。深入实施“五气共治”，倒逼重点管控企业废气达标排放，加强餐饮场所、工地、码头堆场等清洁化管理，加强秸秆资源化利用，完成黄标车淘汰整治，确保全年PM2.5浓度和重污染天数实现“双降”。加强土壤重金属污染防治，开展污染场地治理修复试点。实现固废处置设施县（市）全覆盖。

　　加大“三改一拆”力度。坚持拆改结合、拆用并举，依法治违、长效管护，完成拆改面积1600万平方米、创建2个“基本无违建县（市、区）”。利用好国家对棚户区改造的政策机遇，加快旧住宅区改造，重点做好中心城区杉青闸、文生修道院等地块改造工作。持续深化“四边三化”，巩固“公铁”沿线环境整治成果，全市新增改造绿化面积2.2万亩。

　　加大节能减排力度。健全能源消费总量、单耗和煤炭消费总量“三控”制度，推行用能预算管理，完善用能权指标交易机制。调整能源结构，推进煤改气、油改气以及电能替代等工作，加快太阳能、风能、生物质能利用。建立企业排污双证许可制度，加强对重点企业排污总量监管，严禁新上高污染、高排放项目。大力发展循环经济，加快建设一批示范园区和企业，完成100家企业清洁生产审核。

　　（六）加强社会建设和改善民生

　　促进城乡居民持续增收。落实积极的就业创业政策，实施大学生就业促进计划和创业引领计划，规划建设大学生创客孵化中心6家。加强就业援助，增加公益性就业岗位，促进困难人员就业。加大农村劳动力就业培训力度，新增城镇就业6万人，力争更多的农村劳动力转移到城镇稳定就业。完善社会平均工资指导线制度，推行企业工资集体协商。强化欠薪预警防范和综合处置，加大排查力度，充实工资保证金和应急周转金，坚持打击恶意欠薪、恶意讨薪“两手硬”。

　　完善社会保障制度。加大社会保障扩面工作力度，推进居民医保与职工医保制度的衔接，加快建立城乡一体的失业保险制度，动态调整各类人员社会保障待遇。完善城乡最低生活保障和社会救助工作，做好低收入群体增收帮扶和精准扶贫。加快养老服务体系建设，鼓励发展各类民办养老机构，积极探索医养结合模式，实现居家养老服务照料中心城乡社区全覆盖。加快推进市老年公寓改扩建、市老干部活动中心扩建等项目建设。创新住房保障模式，加快向公共租赁住房货币补贴转变。积极发展红十字和公益慈善事业，完善残疾人社会救助制度，开工建设市残疾人康复中心。

　　推动社会事业普惠均衡发展。大力培育和弘扬社会主义核心价值观，巩固发展全国文明城市创建成果，推进全国“志愿服务模范城”创建。加快国家公共文化服务体系示范区建设，大力实施文化惠民工程，推进农村文化礼堂提质扩面和城市社区文化家园建设，切实做好遗产遗址、历史街区的传承保护和活化利用，加快推动嘉兴博物馆二期、马家浜文化博物馆项目建设。深化教育领域综合改革，建立以县为主学前教育统筹发展机制，加快义务教育优质均衡发展，推进国家特殊教育改革试验区建设，实施职业教育人才培养创新，支持在禾高等院校加快发展。深化公立医院综合改革，推进优质医疗资源下沉基层，抓好分级诊疗，推广诊间结算。做好国家卫生城市复查迎检工作。落实全面两孩政策，启动市妇保院二期建设。实施全民健身工程，提升群众体育、竞技体育和体育产业发展水平。支持工会、共青团、妇联、科协等群团组织创新发展，扎实做好民族宗教、外事侨务、对台事务、统计、档案、史志、气象等工作。

　　加强社会治理创新。深入推进“三社”联动，加大社会组织、专业社工培育力度。健全矛盾纠纷多元化解机制，做好新形势下信访工作。完善公共安全保障体系，强化食品药品监管，全面落实安全生产责任制，坚决遏制重大事故。扎实做好防灾减灾工作。全面开展“七五”普法，完善公共法律服务体系。制定出台户籍制度改革政策，创新推进新居民管理服务。加强互联网综合治理和网络安全维护。强化社会治安综合治理，总结推广“乌镇管家”经验，加强反恐怖能力建设，依法严密防范和严厉打击各类违法犯罪活动。支持驻嘉部队建设，做好国防动员、双拥优抚安置等工作，促进军民融合发展。

　　（七）加强政府自身建设

　　依法履行政府职能。把政府活动全面纳入法治轨道。健全“四张清单一张网”制度，深化简政放权、放管结合、优化服务改革，构建科学合理的政府职责体系。加强政府法制工作，强化与市人大的立法衔接。规范重大行政决策程序，健全公众参与、风险评估、合法性审查等制度。深入推进综合行政执法改革，加快建立统一的政务咨询投诉举报平台和联合执法协调指挥机制。加强和改进行政复议工作。创新公共服务供给方式，编制政府职能向社会组织转移清单。

　　加强行政权力监督。“权力应该被用来限制权力”。自我强化政府受监督意识，自觉接受人大依法监督、政协民主监督、司法监督，加强政府内部层级监督和专门监督，积极接受社会监督和舆论监督，让权力在阳光下运行。全面推进政务公开，加强对重大决策部署落实情况的跟踪审计，加强行政效能监察，加大督查问责力度。

　　加强作风和廉政建设。打铁还需自身硬。深入开展“两学一做”学习教育，把纪律和规矩挺在前面，坚决整治“庸懒散”，破解机关“中梗阻”，决不允许尸位素餐，努力推动作风建设常态化、长效化。健全容错纠错机制，宽容失败，允许试错，有错必改，为改革创新者撑腰鼓劲，为敢于担当者担当。打通联系群众“最后一纳米”，强化第一线服务，把群众的“点赞”作为推动政府工作前进的动力，把群众的“吐槽”作为努力改进政府工作的明确方向。健全廉政风险防控机制，坚决惩处各类腐败行为，坚决做到发现一起，查处一起，绝不姑息。

　　各位代表，时代赋予我们重任，人民寄予我们厚望，唯有实干才能铸就新的辉煌。让我们更加紧密地团结在以习近平同志为总书记的党中央周围，在中共嘉兴市委的坚强领导下，振奋精神、凝心聚力，勇于担当、真抓实干，为实现高水平全面建成小康社会的宏伟目标而不懈奋斗！
